# Nocturnal hypoxemic burden is associated with worsening prognosis of chronic kidney disease in patients with type 2 diabetes

**DOI:** 10.1186/s12933-025-02918-y

**Published:** 2025-08-31

**Authors:** Sarah Driendl, Stefan Stadler, Mathias Baumert, Klaus J. Stark, Iris M. Heid, Jan Pec, Florian Zeman, Adrian Preiss, Carsten A. Böger, Tobias Bergler, Michael Arzt

**Affiliations:** 1 Department of Internal Medicine II, University Hospital Regensburg, Franz-Josef-Strauss-Allee 11, 93053 Regensburg, Germany; 2 Discipline of Biomedical Engineering, School of Electrical and Mechanical Engineering, University of Adelaide, Adelaide, Australia; 3 Department of Genetic Epidemiology, University of Regensburg, Regensburg, Germany; 4 Center for Clinical Studies, University Hospital Regensburg, Regensburg, Germany; 5 Department of Nephrology, University Hospital Regensburg, Regensburg, Germany; 6 Department of Nephrology, Diabetology and Rheumatology, Kliniken Südostbayern, Traunstein, Germany; 7 KfH Kidney Center Traunstein, Transtein, Germany; 8 Medical Clinic III - Nephrology, Hospital Ingolstadt, Ingolstadt, Germany

**Keywords:** Chronic kidney disease, Hypoxia, Nocturnal hypoxemic burden, Kidney failure, Type 2 diabetes, Sleep-disordered breathing, Microvascular disease

## Abstract

**Introduction:**

Lower estimated glomerular filtration rate (eGFR) and more severe albuminuria categories are associated with increased risk for adverse outcomes such as mortality, cardiovascular and kidney outcomes. The aim of the analysis was to evaluate whether nocturnal hypoxemic burden (NHB) is associated with worsening prognosis of CKD in a population with T2D.

**Methods:**

Overnight oximetry data from patients enrolled in the DIACORE (DIAbetes COhoRtE) sleep-disordered breathing sub-study, a prospective cohort study of patients with T2D, was analyzed and NHB as cumulative time spent below 90% oxygen saturation (T90) was quantified. Very-high-risk CKD was defined according to KDIGO risk classification: eGFR < 30 ml/min/1.73 m^2^ regardless of urinary albumin-to-creatinine ratio (uACR); eGFR < 45 ml/min/1.73 m^2^ and uACR > 30 mg albumin/g creatinine; or eGFR < 60 ml/min/1.73 m^2^ and uACR > 300 mg/g. Logistic regression analyses adjusting for known risk factors for CKD prognosis were performed to assess the association between NHB and incident very-high-risk CKD.

**Results:**

The analysis population comprised 857 participants (41% female, mean age 65 years, median diabetes duration 9.0 years, median eGFR 82 ml/min/1.73 m^2^). During follow-up, 72 (8.4%) patients developed very-high-risk CKD, and patients with high T90 significantly more often developed very-high-risk CKD than patients with lower T90 (quartile 4 vs. quartiles 1–3: 15.0 vs. 6.2%, p < 0.001). NHB was significantly associated with an increased incidence of very-high-risk CKD. Patients in the highest quartile of T90 had a 3.0-fold higher risk compared to patients in the lowest quartile, independently of other risk factors for CKD prognosis such as age, sex, waist-hip ratio, hypertension, antihypertensive and lipid-lowering medication, HbA1c, diabetes duration, and eGFR and hemoglobin levels at baseline (OR 2.96, 95% CI (1.24; 7.07), p = 0.014; p for trend 0.013).

**Conclusion:**

We identified NHB as a novel risk factor for worsening CKD prognosis in patients with T2D. Further research is needed to ascertain whether T90 reduction constitutes a clinically meaningful prevention target.

**Trial registration:**

German Clinical Trials Register DRKS00010498.

**Graphical Abstract:**

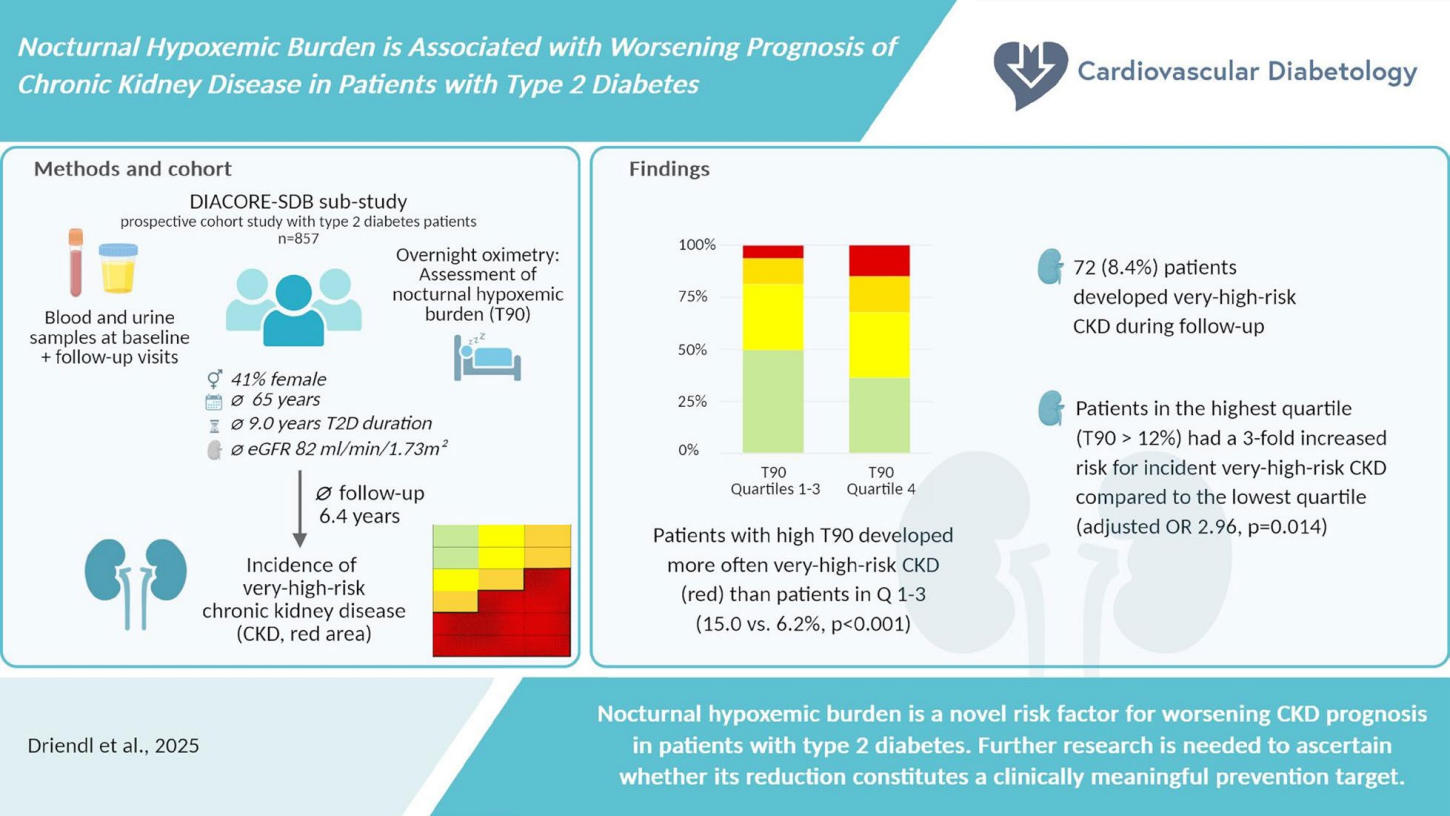

**Supplementary Information:**

The online version contains supplementary material available at 10.1186/s12933-025-02918-y.

## Research Insights

**What is currently known about this topic?**

Chronic kidney disease (CKD) is a common sequela of type 2 diabetes. Identifying risk factors for its progression is crucial.

**What is the key research question?**

Is nocturnal hypoxemia associated with worsening prognosis of CKD?

**What is new?**

Nocturnal hypoxemic burden is a novel risk factor for worsening CKD prognosis in patients with T2D.

**How might this study influence clinical practice?**

Nocturnal hypoxemic burden can be derived at a low cost from widely available pulse oximetry devices. It could serve as a simple prognostic tool.

## Introduction

Type 2 diabetes (T2D) is one of the leading causes of death and disability worldwide [1]. It is highly prevalent, affecting 529 million people worldwide in 2021, with rising numbers globally [1]. T2D is a major risk factor for chronic kidney disease (CKD) [2], which is defined by the Kidney Disease Improving Global Outcomes (KDIGO) initiative as abnormalities of kidney structure or function, persisting ≥ 3 months, with implications for health [3]. CKD is projected to be the fifth leading cause of death by 2040 [4]. Over 40% of individuals with diabetes develop CKD, many progressing to kidney failure requiring dialysis or transplantation [5], contributing to a substantial global health and economic burden [6].

**Fig. 1:**
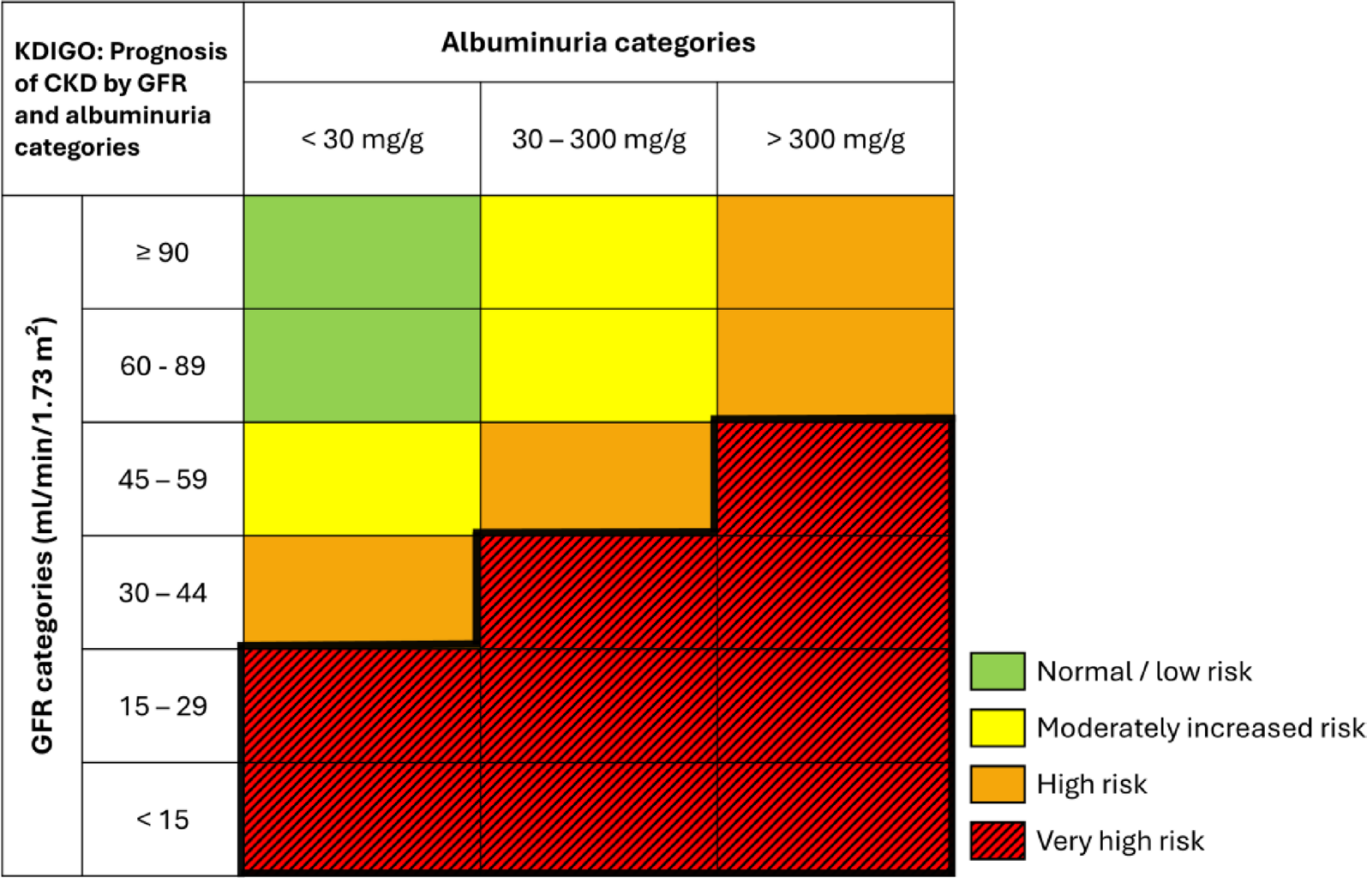
Heatmap indicating risk prognosis of chronic kidney disease (CKD) by eGFR and albuminuria categories. Risk prognosis includes risk with worsening CKD for 10 adverse outcomes: kidney outcomes such as kidney failure requiring replacement therapy and acute kidney injury, cardiovascular outcomes such as cardiovascular mortality, coronary heart disease, stroke, heart failure, atrial fibrillation, and peripheral artery disease, and other health outcomes such as all-cause mortality, and any hospitalization. Figure adapted from the KDIGO 2024 Clinical Practice Guideline for the Evaluation and Management of Chronic Kidney Disease [3]

CKD severity is classified by kidney excretory capacity (estimated glomerular filtration rate, eGFR) and albuminuria [3]. A recent meta-analysis of 114 cohorts (> 27 million individuals) demonstrated that lower eGFR and higher urinary albumin-to-creatinine ratio (uACR) are independently associated with 10 adverse outcomes such as mortality, kidney, cardiovascular, and other health outcomes, with risks increasing progressively across worsening categories [7]. The KDIGO 2024 guideline visualized these risks using “heatmaps”, color-coding the escalating risk for the above-named outcomes (Fig. 1) [3]. Understanding risk factors contributing to CKD progression is therefore crucial to identify high-risk patients early.

Nocturnal hypoxemia has been proposed as one such risk factor. Experimental studies indicate that chronic intermittent hypoxia contributes to renal damage and dysfunction by promoting renal hypoperfusion and tissue hypoxia during wakefulness and sleep [8]. In animal models, long-term hypoxia reduced eGFR [9], and in humans, sleep-disordered breathing (SDB) has been linked to a higher incidence of end-stage kidney disease [10, 11]. However, clinical evidence on the impact of nocturnal hypoxemia on the prognosis of CKD in T2D is scarce and limited to small populations including participants with and without T2D with limited follow-up time [12, 13].

The aim of the analysis was to evaluate whether nocturnal hypoxemic burden (NHB) is associated with worsening prognosis of CKD in a large population with T2D.

## Methods

### Study population

The DIACORE (DIAbetes COhoRtE) study is a prospective cohort study of patients with T2D aiming to identify mechanisms involved in the development and progression of T2D complications. All patients with T2D living in the region around the study center were invited to participate. This was achieved by means of written invitations being sent to all T2D patients in the records of local diabetologists, general practitioners and health insurance companies [14]. The recruitment process has been described previously [14]. Inclusion criteria were the ability to provide written informed consent, age ≥ 18 years, self-reported Caucasian ethnicity and prevalent T2D [14]. Exclusion criteria included haemochromatosis, acute infection or fever, chronic viral hepatitis, HIV infection, autoimmune disease potentially affecting kidney function (e.g. systemic lupus erythematodes), and ongoing chronic renal replacement therapy at baseline (hemodialysis, peritoneal dialysis or transplantation) [14]. Participants from the study site in Regensburg who did not use positive airway pressure therapy were eligible to participate in the DIACORE-SDB sub-study. Polygraphy was conducted between November 2011 and October 2015. A standardized questionnaire was used to evaluate alcohol intake (number of drinks per week), smoking status (current, former or never), socioeconomic status (according to the Robert Koch Institute, Germany [15], subdivided into four groups ranging from 1 (lowest) to 4 (highest) and including educational level, professional qualification and income), and physical activity (defined as light activity at least three times per week). Blood pressure was determined via repeated measurement: three measures were performed, and the last two were averaged [14]. The DIACORE study and its protocol have been approved by the University of Regensburg Ethics Committee (vote 06-139) and were in accordance with the Declaration of Helsinki. Participation in the DIACORE study was based on the patient`s written consent. The reporting adhered to the STROBE checklist for observational studies. The study is registered with the German Clinical Trials Register (DRKS00010498).

### Quantification of nocturnal hypoxemic burden

Unattended polygraphy was performed over one night at the participants` residence using a validated 2-channel ambulatory monitoring device [16] recording nasal flow and pulse oximetry (ApneaLink^®^, ResMed), as described previously [17]. Participants were instructed in a standardized manner on how to use the device by trained personnel. Oximetry signals were extracted for further processing using a fully automated and custom-made computer algorithm programmed in MATLAB^®^ (MathWorks^®^, Natick, MA, USA), as described previously [18]. Signal artifacts were automatically detected and excluded based on empirical criteria [18]. Nocturnal hypoxemic burden was defined as cumulative recording time spent at SpO_2_ levels below 90% (T90). As no clinical cut-off exists, T90 was grouped by quartiles, and cut-offs from literature were used additionally, stratifying the cohort by T90 > 12 min [19] or T90 > 10% [20]. The oxygen-desaturation index (ODI) measures the mean number of respiratory events per hour where blood oxygen level dropped by 4% compared to the immediately preceding basal value. The apnea–hypopnea index (AHI) was calculated as the mean number of apnea and hypopnea events per hour of recording time. The standard settings of the monitoring device were used to define apnea, hypopnea, and desaturation [21]. All patients were informed about the results of SDB monitoring, but further diagnostics and treatment were not part of the DIACORE protocol.

### Follow-up

Patients were followed up every two years until December 2021. The participants completed a standardized questionnaire and underwent a physical examination at every visit [14]. Blood samples were drawn after the patients had rested in a seated position for at least 15 min at every study center visit [14]. Following the interview, the patients were asked to provide a spot midstream urine sample [14]. Positive airway pressure treatment use was evaluated using standardized questionnaires during follow-up as part of the DIACORE protocol.

### Kidney function measures

All patients had serum creatinine measurements for eGFR using the 2009 Chronic Kidney Disease Epidemiology Collaboration (CKD-EPI) creatinine equation. The categories of eGFR were 90 ml/min/1.73 m^2^ or higher, 60–89 ml/min/1.73 m^2^, 45–59 ml/min/1.73 m^2^, 30–44 ml/min/1.73 m^2^, 15–29 ml/min/1.73 m^2^, and less than 15 ml/min/1.73 m^2^, according to KDIGO guidelines [3]. The urinary albumin-to-creatinine ratio (uACR) was calculated as urinary albumin (mg)/urinary creatinine (g) and classified into categories of less than 30 mg/g (A1), 30 to 299 mg/g (A2), and 300 mg/g or greater (A3). Data on initiation of treatment for kidney failure (kidney replacement therapy, defined as self-reported dialysis or kidney transplantation) and on mortality were ascertained by self-reported history and validated from medical records and direct contact with the patients’ treating physicians [14]. Medical documentation was requested up to three times from the patients’ treating physicians. If a patient-reported end-point could not be confirmed by available documentation or if adequate medical documentation was not available, then that endpoint was coded as “not validated” [14]. The last study visit with available serum and urine samples was analyzed for the present analysis. The KDIGO classification consists of four categories to categorize CKD prognosis based on risk of adverse outcomes such as all-cause and cardiovascular mortality, renal and cardiovascular outcomes, as described above [3]: (1) normal or low risk, (2) moderately increased risk, (3) high risk, and (4) very high risk. Very-high-risk CKD is defined as eGFR < 30 ml/min/1.73 m^2^ regardless of uACR; eGFR < 45 ml/min/1.73 m^2^ and uACR > 30 mg albumin/g creatinine; or eGFR < 60 ml/min/1.73 m^2^ and uACR > 300 mg/g. Risk categories are visualized in Fig. 1, adapted from the KDIGO 2024 Clinical Practice Guideline for the Evaluation and Management of CKD [3].

### Outcomes

We considered two major endpoints: (1) the incidence of very-high-risk CKD among patients not in this category at baseline during follow-up (Fig. 1, dark red area), and (2) the incidence of kidney failure defined as a composite endpoint of a decreased eGFR of at least 40% from baseline, end-stage kidney disease, or death from renal causes. As both eGFR and albuminuria are part of the classification of CKD risk class, for the first outcome, only patients who provided complete blood and urine samples at baseline as well as concurrent blood and urine samples during follow-up were included (Figure S1). For the second outcome, all patients who provided blood samples at baseline and during follow-up were analyzed (Figure S1).

### Statistical analysis

Descriptive data are presented as mean (± standard deviation) and median [interquartile range] or number (percentage) for categorical variables. We conducted multivariable binary logistic regression analyses adjusting for known risk factors for progression of chronic kidney disease, i.e., sex, age (years), waist-hip ratio, arterial hypertension (yes/no, defined as systolic blood pressure ≥ 120 mmHg, according to current guidelines for patients with CKD [3]), antihypertensive and lipid-lowering medication, duration of T2D (years), glycated hemoglobin (HbA1c, mmol/mol), hemoglobin levels at baseline [g/dl], and eGFR strata at baseline. With a maximum of 2.3% missing values, we used complete case analysis to handle the missing data. Effect modification by sex, age (≤ 65 or > 65 years), and diabetes duration (< 5 or ≥ 5 years) was explored via stratified subgroup analyses and tested for interaction. Results are presented as odds ratio (OR) estimates with a 95% confidence interval (CI). P values < 0.05 were considered statistically significant. Data were analyzed using the SPSS statistical software package (SPSS 28.0 IBM SPSS Statistics, Armonk, New York, USA).

## Results

### Patient characteristics

Of the 1491 patients in the DIACORE-SDB sub-study, 313 patients were excluded due to incomplete polygraphy or follow-up, or missing blood samples during follow-up (Supplemental Figure S1). 270 patients did not provide urinary samples at baseline or during follow-up and, therefore, were excluded from the analysis of the first outcome (Supplemental Figure S1), and 52 patients had very-high-risk CKD at baseline.

At baseline, the mean age of the cohort (n = 857) was 65 ± 9 years, and 350 (41%) were female (Table 1). 420 (49%) were obese (BMI ≥ 30 kg/m^2^), and the mean waist-hip-ratio was 0.95. The median glycated hemoglobin level was 6.5% (48 mmol/mol), and the median duration of T2D was 9.0 years. Due to recruiting starting in 2011, none of the patients received SGLT2 inhibitors. Kidney function was preserved in most patients with a median eGFR of 82 ml/min/1.73 m^2^ and a median uACR of 8.8 mg/g (= G2A1). At baseline, 585 (68%), 203 (24%) and 69 (8%) were in the CKD risk categories of normal/low, moderately increased and high risk, respectively. The median nocturnal hypoxemic burden was 3.4% or 14 min. In 56 (6.5%) patients, oxygen saturation was never below 90%. Long T90 durations were evident across the SDB severity spectrum [22, 23]. In a subset of 285 patients, nocturnal hypoxemic burden was additionally assessed at visit 2. The mean T90 was 10.01% at baseline and 10.38% at visit 2. There was no statistical difference between these two variables (p = 0.880). The stability of exposure values across the two time points was further assessed using a two-way mixed Intraclass Correlation Coefficient (ICC). Average-measure ICC was 0.640 (p < 0.001), indicating moderate stability.

**Table 1:** Baseline characteristics of the entire cohort and according to T90

Variables	Entire cohort	T90 Q 1/2/3	T90 Q 4
n (%)	857	643 (75)	214 (25)
Female sex, n (%)	350 (41)	274 (43)	76 (36)
Age [years]	65 ± 9	65 ± 9	67 ± 8
BMI [kg/m^2^]	30.7 ± 5.1	30.2 ± 5.0	32.2 ± 5.1
Waist-hip ratio	0.95 ± 0.08	0.94 ± 0.08	0.97 ± 0.08
Former or current smokers, n (%)	487 (57)	358 (56)	129 (60)
High alcohol intake, n (%)	250 (30)	179 (28)	71 (34)
Physical activity, n (%)	380 (45)	300 (47)	80 (38)
Socioeconomic status, n (%)			
Low	229 (27)	165 (28)	64 (30)
Lower–middle	432 (51)	327 (52)	105 (50)
Upper–middle	115 (14)	88 (14)	27 (13)
High	66 (8)	51 (8)	15 (7)
eGFR** [**ml/min/1.73 m^2^]	82 [68; 93]	81 [68; 95]	74 [63; 88]
eGFR category [ml/min/1.73 m^2^], n (%)			
≥ 90 (stage 1)	279 (33)	226 (35)	53 (25)
60–89 (stage 2)	457 (53)	338 (53)	119 (56)
30–59 (stage 3)	121 (14)	79 (12)	42 (20)
< 30 (stage 4/5)	–	–	–
uACR [mg/g]	8.8 [4.2; 24.7]	8.3 [3.9; 21.2]	11.0 [5.7; 35.7]
uACR category, n (%)			
< 30 mg/g	677 (79)	524 (82)	153 (72)
30–300 mg/g	180 (21)	119 (19)	61 (29)
> 300 mg/g	–	–	–
CKD risk stage, n (%)			
Normal/low	585 (68)	461 (72)	124 (58)
Moderate	203 (24)	140 (22)	63 (29)
High	69 (8)	42 (7)	27 (13)
HbA1c [mmol/mol (%)]	48 (6.5) [43 (6.1); 55 (7.2)]	48 (6.5) [43 (6.1); 54 (7.1)]	49 (6.6) [44 (6.2); 56 (7.3)]
T2D duration [years]	9.0 [5.2; 14.7]	8.9 [5.1; 14.6]	9.9 [5.4; 14.9]
Any glucose-lowering medication, n (%)	731 (85)	546 (86)	185 (88)
Insulin, n (%)	229 (27)	160 (26)	69 (33)
Oral antidiabetic drug, n (%)	663 (77)	483 (76)	171 (81)
Systolic BP [mmHg]	138 ± 17	138 ± 17	139 ± 18
Diastolic BP [mmHg]	75 ± 10	75 ± 9	75 ± 10
Systolic BP > 120 mmHg, n (%)	737 (86)	553 (86)	184 (86)
Cardiovascular conditions, n (%)			
Hypertension	344 (40)	263 (41)	81 (38)
Coronary artery disease	146 (17)	106 (17)	40 (19)
Stroke	42 (5)	25 (4)	17 (8)
Antihypertensive drugs, n (%)	663 (77)	477 (74)	186 (87)
LDL [mg/dl]	118 ± 36	119 ± 35	113 ± 36
HDL [mg/dl]	54 ± 16	54 ± 15	53 ± 16
Lipid lowering drugs, n (%)	402 (47)	291 (45)	111 (52)
Hb [g/dl]	14.3 ± 1.2	14.3 ± 1.2	14.4 ± 1.3
Mean SpO_2_ [%]	92 ± 2	93 ± 1	90 ± 1
Min SpO_2_ [%]	81 [78; 83]	82 [80; 85]	79 [74; 82]
T90 [%/TRT]	3.4 [0.5; 11.8]	1.4 [0.2; 4.8]	26.1 [16.6; 46.2]
ODI [events/h]	6 [3; 14]	5 [2; 12]	10 [4; 20]
AHI [events/h]	9 [5; 18]	8 [5; 186]	13 [7; 24]
Excessive daytime sleepiness, n (%)	64 (8)	44 (7)	20 (10)
PAP use at follow-up, n (%)	85 (10)	63 (10)	22 (10)
Follow-up time [years]	6.4 [5.2; 8.2]	6.5 [5.2; 8.2]	6.4 [5.2; 8.1]

Results are provided as mean ± standard deviation or median [interquartile range].

AHI, apnea–hypopnea index; BMI, body-mass index; BP, blood pressure; CKD, chronic kidney disease; Hb, hemoglobin;HbA1c, hemoglobin A1c; HDL, high-density lipoprotein; LDL, low-density lipoprotein; ODI, oxygen-desaturation index; PAP, positive airway pressure; SpO_2_, arterial oxygen saturation; T90, night-time spent with oxygen saturation < 90%; TRT, total recording time; uACR, urinary albumin-to-creatinine ratio.

High alcohol intake, defined as ≥ 3/drinks per week; excessive daytime sleepiness, defined as Epworth Sleepiness Scale ≥ 11; physical activity, defined as light activity > 2 times/week; hypertension, defined as blood pressure ≥ 140/90 mmHg.

Socioeconomic status was available in 842 patients. PAP status was available in 790 patients. Information on antidiabetic drugs available in 845 patients, including insulin, biguanides, sulfonylureas, glitazones, DPP-4 inhibitors, GLP-1 receptor agonists, alpha-glucosidase inhibitors.

### Nocturnal hypoxemic burden and the incidence of very-high-risk CKD

**Fig. 2:**
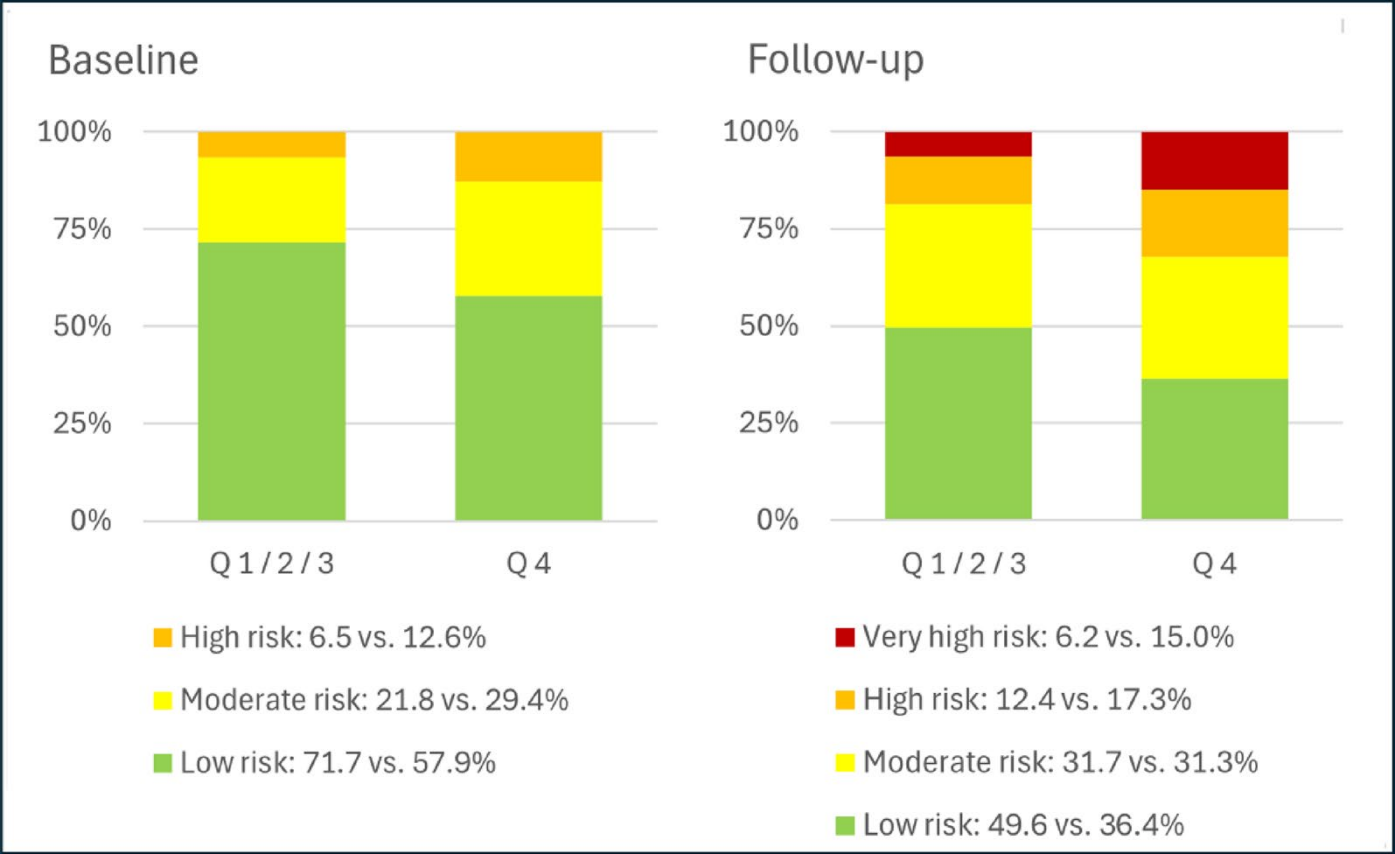
CKD risk classes at baseline and follow-up according to patients with lower T90 (quartiles (Q) 1, 2, and 3) and high T90 (Q 4). Patients in the highest T90 quartile exhibited poorer risk classes than those with lower T90 values

**Fig. 3:**
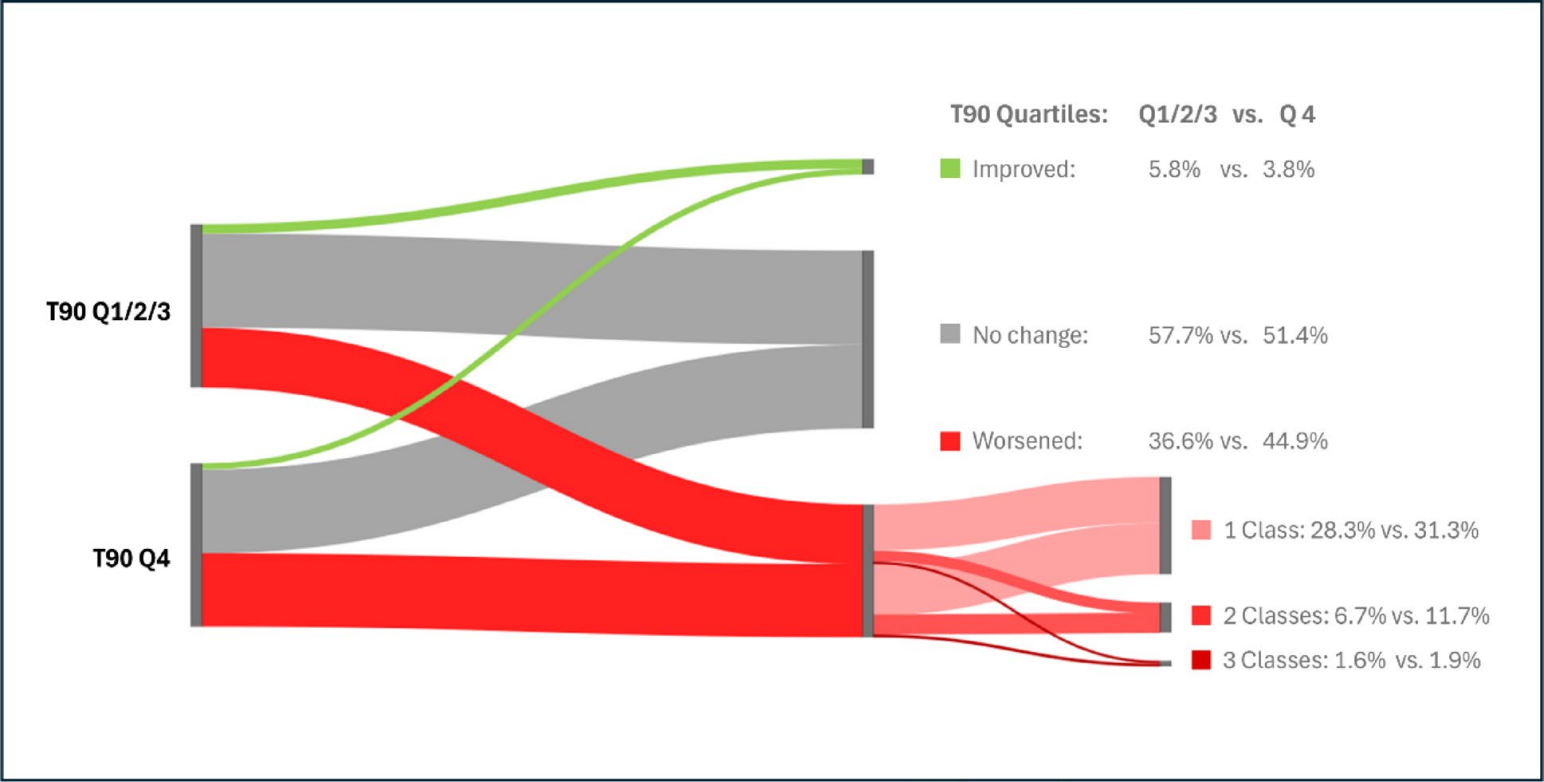
Alluvial diagram of CKD risk class changes between baseline and follow-up according to T90 quartiles 1 to 3 and quartile 4. In patients with a high T90 (quartile 4), an improvement in CKD risk class was less frequent, and a worsening in risk class was observed more often

72 (8.4%) patients developed very-high-risk CKD during the follow-up. Very-high-risk CKD was significantly more frequent in patients with higher T90 (quartile 4: 32 (15.0%) versus quartile 1: 10 (4.7%), p < 0.001). Figure 2 visualizes the risk classes at baseline and during follow-up according to patients in T90 quartiles 1 to 3 versus quartile 4. Patients in quartile 4 developed significantly more often very-high-risk CKD during follow-up than patients in quartiles 1 to 3 (6.2 vs 15.0%, p < 0.001). Figure 3 illustrates the difference in CKD risk class change between baseline and follow-up according to T90 groups. Patients with a high T90 (quartile 4) less often had an improvement in CKD risk class, had a higher frequency of deterioration of risk class, and, within this category, a greater occurrence of worsening of 2 or 3 risk classes compared with patients in T90 quartiles 1 to 3.

Binary logistic regression analysis showed increasing odds of developing very-high-risk CKD for increasing T90 quartiles in univariable and multivariable models (adjusted P for trend 0.013, Table 2). Patients in the fourth quartile, which implied T90 > 11.8%, had a threefold increased risk compared to patients in quartile 1 (T90 < 0.5%), independently of other risk factors for worsening CKD such as age, sex, waist-hip ratio, hypertension, antihypertensive and lipid-lowering medication, HbA1c, diabetes duration, eGFR at baseline, and hemoglobin levels at baseline (Table 2). The application of supplementary cut-offs from the literature (T90 > 12 min [19] or T90 > 10% [20]) yielded consistent results (Supplemental Table S1).

**Table 2:** Binary logistic regression model assessing the association between T90 quartiles and the incidence of very-high-risk CKD during follow-up, each quartile compared to the lowest quartile 1

	OR (95% CI), univariable	P value	OR (95% CI), multivariable*	P value
Quartile 1	1.00 (Ref.)		1.00 (Ref.)	
Quartile 2	1.54 (0.68; 3.52)	0.300	1.65 (0.64; 4.22)	0.298
Quartile 3	1.54 (0.68; 3.52)	0.300	1.56 (0.61; 3.99)	0.351
Quartile 4	3.60 (1.72; 7.54)	< 0.001	2.96 (1.24; 7.07)	0.014
P for trend		< 0.001		0.013

*Adjusted for age, sex, waist-hip ratio, systolic blood pressure < vs. ≥ 120 mmHg, antihypertensive medication (renin–angiotensin–aldosterone system blockers, calcium channel blockers, betablockers), lipid-lowering medication, HbA1c, diabetes duration, eGFR strata at baseline, and hemoglobin level at baseline

An additional sensitivity analysis in a subgroup of 651 patients with at least five years of follow-up was conducted to eliminate the possibility that an insufficient follow-up period would result in an underestimation of endpoints. The results were similar (OR 3.44, 95% CI (1.22; 9.72), p = 0.020; Supplemental Table S2).

To explore potential heterogeneity in the association between nocturnal hypoxemia and the incidence of very-high-risk CKD, we performed subgroup analyses stratified by sex, age, and diabetes duration. Females with a high T90 (quartile 4) were at 2.8-fold greater risk for incidence of very-high-risk CKD than females with lower T90 independently of other risk factors (Supplemental Table S3). Males with T90 above the median did not exhibit significantly increased risk, but this sex-specific difference was not statistically significant (interaction term P = 0.493). For age and diabetes duration, older patients > 65 years old and patients with diabetes duration > 5 years exhibited increased risk, but these differences also were not significantly different between the groups (interaction terms p = 0.267 and p = 0.432, respectively; Supplemental Table S3).

### Nocturnal hypoxemic burden and the incidence of kidney failure

**Fig. 4:**
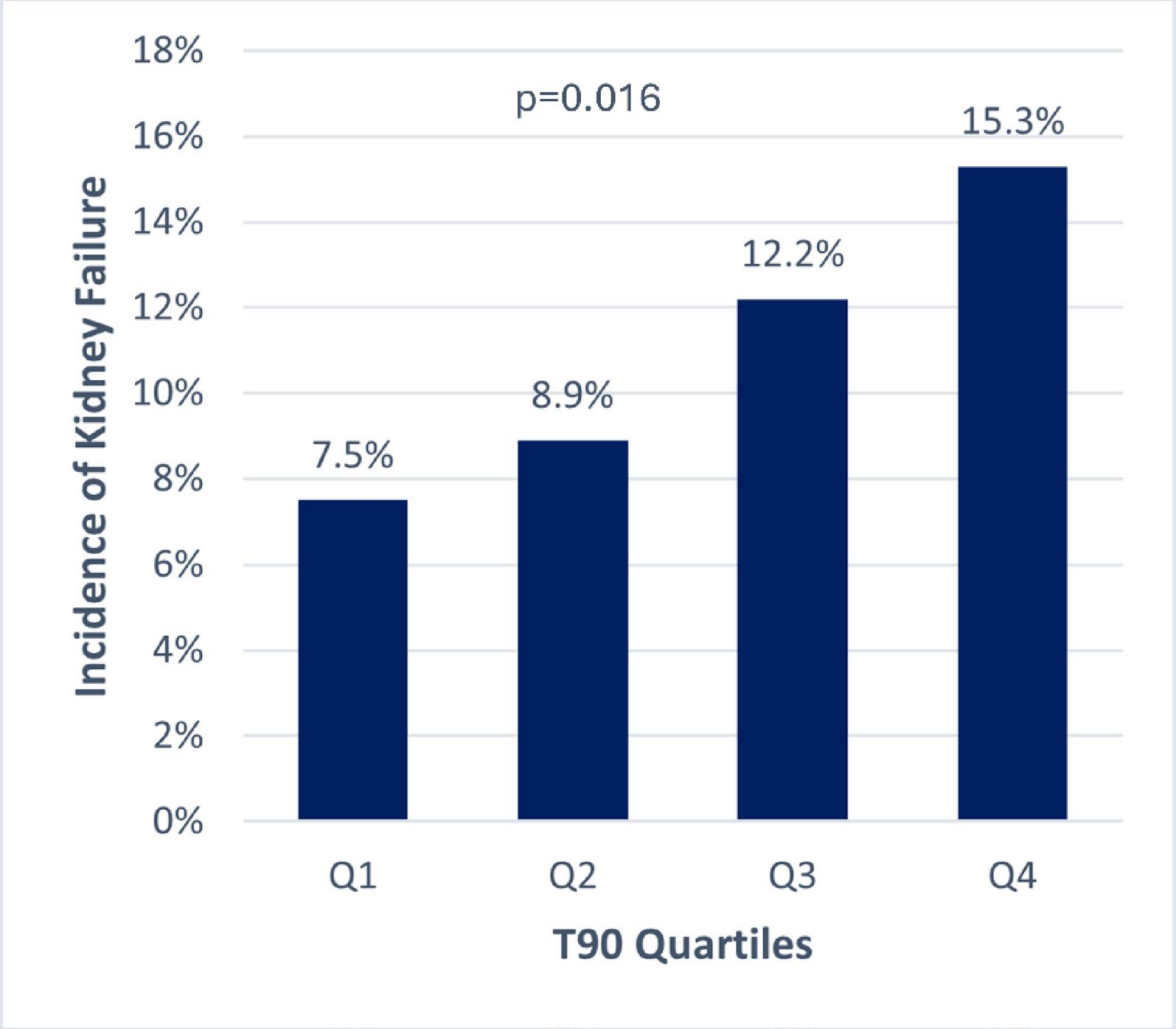
Incidence of kidney failure according to T90 quartiles. The incidence of kidney failure was significantly higher in higher quartiles of T90 compared to lower quartiles

Data from 1179 patients with available blood sample during follow-up were available to analyze the endpoint of kidney failure (Supplemental Figure S1). Baseline characteristics of this cohort are presented in Supplemental Table S4, showing no major differences from the subset (Table 1). 130 (11%) of the patients developed kidney failure during follow-up. Of these, 13 had end-stage kidney disease, and two died of renal cause. Patients with higher T90 significantly more often developed kidney failure than those with lower T90 (Fig. 4). In univariable regression analysis, T90 was significantly associated with incident kidney failure, but this association became insignificant after adjusting for confounders (Table 3).

**Table 3:** Binary logistic regression analyses of the association between nocturnal hypoxemic burden and the incidence of kidney failure comparing each quartile of T90 versus the first quartile in the entire and the stratified cohort ≤ 65 or > 65 years

	Entire cohort	Age ≤ 65 years	Age > 65 years
Quartile 1	1.00 (Ref.)	1.00 (Ref.)	1.00 (Ref.)
Quartile 2	0.96 (0.52; 1.78)	1.83 (0.43; 7.76)	0.51 (0.26; 0.98)
Quartile 3	1.25 (0.70; 2.24)	3.95 (1.04; 15.09)	0.54 (0.29; 1.01)
Quartile 4	1.28 (0.71; 2.28)	3.52 (0.89; 13.95)	0.75 (0.41; 1.37)
P for trend	0.275	**0.027**	0.719

Depicted as OR and 95% CI. Analyses adjusted age, sex, waist-hip-ratio, hypertension, HbA1c, diabetes duration, and eGFR at baseline. In stratified analyses not adjusted for age.

To identify subgroups where high T90 presents a greater risk, we stratified the cohort according to sex, age, and diabetes duration. While sex and diabetes duration did not mediate the relationship between T90 and outcomes (Supplemental Table S5), higher T90 was associated with incident kidney failure in younger patients (≤ 65 years), but not in older patients. When comparing each quartile versus the first quartile in the younger age strata, patients in quartiles 3 and 4 had substantially increased odds compared to those in quartile 1 (HR 3.95 and 3.52, respectively, p for trend 0.027; Table 3). Dichotomizing the cohort by the median, patients ≤ 65 years old and T90 above the median had a 2.3-fold increased risk for the incidence of kidney failure compared to T90 below the median (Supplemental Table S5). The interaction between dichotomized nocturnal hypoxemic burden and age groups was not statistically significant (P = 0.111), though a trend towards effect modification was observed when comparison between Q4 and Q1 was taken age-group interaction into account (P for interaction 0.057).

## Discussion

Our study identified nocturnal hypoxemic burden as a novel risk factor for worsening CKD prognosis in T2D. In a large population of T2D patients, patients in the highest quartile of T90 had a threefold increased risk for worsening prognosis of CKD compared to those in the lowest quartile, independently of known risk factors such as age, sex, waist-hip ratio, hypertension, antihypertensive and lipid-lowering medication, HbA1c, diabetes duration, eGFR and hemoglobin levels at baseline. Moreover, kidney failure was more frequent in patients with higher T90, and younger patients tended to be at increased risk.

### Incidence of very-high-risk CKD

Evidence on the impact of nocturnal hypoxemia on the prognosis of CKD is scarce and limited to mixed cohort studies, including patients with and without T2D, with limited follow-up time. In a retrospective cohort study of 120 non-obese patients in CKD stages 3 and 4 (24% with T2D), T90 was significantly associated with kidney function decline after one-year follow-up [12]. A study in 858 patients with suspected sleep apnea (20% with T2D) showed that T90 > 12% was associated with a 2.9-fold increased odds for accelerated kidney function loss, defined as a decline in eGFR ≥ 4 ml/min/1.73 m^2^ per year after a mean follow-up of 2.1 years compared to patients with lower NHB [13]. A prospective observational study on 90 extremely obese patients with T2D (mean BMI 46.8 kg/m^2^) observed that T90 was inversely correlated with eGFR, and T90 was four times greater in patients with CKD than in patients without CKD [24].

Meta-analyses show that sleep-disordered breathing increases the odds for CKD as well as for higher albuminuria/proteinuria and lower eGFR [25–27]. Yet, evidence increasingly shows that hypoxia itself is a substantial factor that promotes CKD progression. In response to hypoxia, tubular epithelial cells exhibit changes such as cell death or atrophy, maladaptive repair, or metabolism switch, and the consequent production of different bioactive molecules drives interstitial inflammation and fibrosis [28]. Hypoxia per se was associated with the development of proteinuria, increased renal vimentin expression, a marker of tubular damage, and infiltration of inflammatory cells in a rat model of 2,4-dinitrophenol treatment, leading to increased renal oxygen consumption and thus to renal tissue hypoxia [29]. Remarkably, these effects were seen without hypertension, hyperglycemia or oxidative stress [29]. Moreover, hypoxia activates the sympathetic nervous system and the renin–angiotensin–aldosterone system (RAAS) [30]. The decline in effective renal plasma flow in response to angiotensin II was less in patients with severe hypoxemia than those with moderate hypoxemia and control subjects, reflecting augmented renal RAAS activity [30].

In the present study, women with high T90 did not exhibit a statistically significant increased risk for the development of very-high-risk CKD compared to men with high T90. Large studies showed that the male sex is associated with enhanced risk for the progression of acute kidney injury and CKD in human and animal models [31]. Research in both human and animal models has demonstrated that estrogens exhibit renoprotective properties in acute kidney injury and attenuate the risk of developing kidney failure in females by promoting renal vasodilation and reducing inflammation, fibrosis, oxidative stress, and cell death [32]. However, other studies observed that in diabetes, women were more susceptible to changes in eGFR, with obesity and vascular stiffness positively correlating with an overall decline in GFR [33]. Given the mean age of 65 years in the present study, it is plausible that the renoprotective effects of estrogen are also diminished. A large meta-analysis of ten studies with more than 5 million participants showed that the excess risk for end-stage kidney disease was higher in women with diabetes than in diabetic men, suggesting that CKD progression accelerates in women with diabetes [34]. In the present study, the hemoglobin levels at baseline were lower in women than in men (13.7 versus 14.7 g/dl, respectively, P < 0.001), which could also make women more susceptible to the deleterious effects of intensified NHB [28]. Yet, study findings are heterogeneous and more research, especially in T2D, is needed to assess the possible sex differences and their mechanisms.

### Incidence of kidney failure

Whereas patients with higher NHB significantly more often had kidney failure during follow-up, no significant association was observed after adjustment in multivariable regression models. Yet, increased odds were observed for patients within the highest quartile of T90 vs. the lowest quartile (OR 1.28, 95% CI (0.71; 2.28)) but did not reach statistical significance. To our knowledge, no prior studies have assessed the association between NHB and kidney failure. However, two studies observed an association between sleep apnea and end-stage kidney disease. In a retrospective cohort study of 4,674 patients with newly diagnosed sleep apnea and 23,370 controls without sleep apnea, patients with sleep apnea had a 2.2-fold increased risk for developing end-stage kidney disease compared to controls after a median follow-up of 5.2 years [10]. Another retrospective population-based cohort study found similar results with an increased risk of incident end-stage kidney disease with an adjusted hazard ratio of 1.3 for 67,359 patients with obstructive sleep apnea compared to 336,795 enrollment-matched controls without obstructive sleep apnea during follow-up [11]. As both studies used diagnosis codes defined by the International Classification of Diseases (ICD) from insurance databases to assess the diagnoses of sleep apnea, no information on SDB severity or nocturnal hypoxemia is provided.

Younger patients with severely increased NHB had an increased risk for incident kidney failure compared to those with low NHB, whereas no increased odds were observed in older patients. Age is a non-modifiable risk factor for CKD with age leading to a decline in kidney capacity attributable to irreversible loss of parenchymal cells and entire nephrons [2]. Other comorbidities also related to CKD, such as arterial hypertension, are more prevalent in older patients, so it is possible that in these patients, other risk factors for the progression of CKD predominate. This finding highlights the significance of timely identification of patients at risk, thereby enabling close monitoring and early intervention. Yet, more research is needed to investigate the impact of nocturnal hypoxemia on kidney failure and subgroups at increased risk.

### Key findings in context

The endpoint of kidney failure as defined in the present study, is a widely accepted endpoint in large studies [35, 36], and only needs blood samples to determine eGFR in addition to the clinical information. In contrast, there are few studies examining the incidence of very-high-risk CKD as a specific endpoint in literature. In the present study, NHB was associated with very-high-risk CKD, but not with kidney failure in the cohort of T2D patients. Given the enormous clinical relevance of this parameter with its great implications for the prognosis of various outcomes, as recently demonstrated in a substantial meta-analysis encompassing over 27 million individuals [7], the study highlights the necessity to investigate different aspects of CKD. The identification of risk factors that influence the progression of CKD thus leading to worsening prognosis is of significant interest.

### Limitations

The study’s findings must be interpreted in the context of several limitations. First, the observational design of the study precludes causal inference, and residual confounding from unmeasured variables cannot be ruled out despite adjusting for the main known risk factors for CKD. Second, the NHB measurement is based on total recording time rather than actual sleep duration. This may attenuate the precision of sleep-related effects and thereby introducing a conservative bias and consequently leading to an underestimation of the true association. Third, data collection on NHB and laboratory analyses including eGFR are based on single measurements at baseline. Temporal changes during follow-up could have resulted in exposure misclassification, potentially attenuating the observed associations and limit causal inference. Forth, as the study protocol did not enable us to verify chronicity, each patient was categorized at the last study visit using eGFR and uACR at a single point, which deviates from the current KDIGO guidelines defining CKD as reduced kidney function for at least 3 months [3]. Lastly, the study enrolled only Caucasian patients and consequently, our findings may not be generalizable to other ethnicities.

## Conclusion

This present study is the first to identify nocturnal hypoxemic burden as a novel risk factor for worsening CKD prognosis in T2D. NHB can be derived at a low cost from widely available pulse oximetry devices, including consumer wearables, which are increasingly popular and enable large-scale population screening. Thus, NHB may serve as a simple tool to identify T2D patients at increased risk. Further research is required to ascertain underlying pathophysiological mechanisms and to determine whether its reduction constitutes a clinically significant prevention or treatment target.

## Supplementary Information

Below is the link to the electronic supplementary material.

Supplementary Material 1

## Data Availability

The patient data generated during the study is confidential. The dataset analyzed in the current study is available from the corresponding author upon reasonable request.
