# Supplementary material for: Nocturnal hypoxemic burden is associated with worsening prognosis of chronic kidney disease in patients with type 2 diabetes
Source: Cardiovasc Diabetol. 2025 Aug 31;24:354. doi: 10.1186/s12933-025-02918-y (PMC12400664; doi:10.1186/s12933-025-02918-y)

**Supplemental Tables**

Supplemental Table S1: Additional analyses assessing the association between nocturnal hypoxemic burden and the incidence of very high risk CKD for different cut-offs.

|  | OR (95% CI), univariable | P value | OR (95% CI), multivariable* | P value |
| --- | --- | --- | --- | --- |
| T90 continuous, % | 1.01 (1.00; 1.03) | **0.010** | 1.01 (1.00; 1.02) | 0.171 |
| T90 continuous, minutes | 1.00 (1.00; 1.01) | **0.006** | 1.00 (1.00; 1.01) | 0.159 |
| T90 Q1/2/3 vs. 4 | 2.65 (1.62; 4.34) | **<0.001** | 2.08 (1.18; 3.68) | **0.012** |
| T90 > vs. ≤ 12 minutes | 2.15 (1.28; 3.63) | **0.004** | 1.98 (1.08; 3.62) | **0.026** |
| T90 > vs. ≤ 10% | 2.93 (1.79; 4.77) | **<0.001** | 2.50 (1.41; 4.42) | **0.002** |

* Adjusted for age, sex, waist-hip ratio, systolic blood pressure < vs. ≥120 mmHg, antihypertensive medication (renin-angiotensin-aldosterone system blockers, calcium channel blockers, betablockers), lipid-lowering medication, HbA1c, diabetes duration, eGFR strata at baseline, and hemoglobin level at baseline.

Supplemental Table S2: Binary logistic regression model assessing the association between T90 quartiles and the incidence of **very-high-risk** CKD during follow-up, each quartile compared to the lowest quartile 1 in 651 patients with follow-up ≥ 5 years.

|  | **Univariable** | **P value** | **Multivariable*** | **P value** |
| --- | --- | --- | --- | --- |
| **Quartile 1** | 1 (Ref.) |  | 1 (Ref.) |  |
| **Quartile 2** | 1.85 (0.67; 5.11) | 0.239 | 2.12 (0.69; 6.48) | 0.188 |
| **Quartile 3** | 2.12 (0.77; 5.89) | 0.148 | 2.03 (0.66; 6.28) | 0.218 |
| **Quartile 4** | 4.63 (1.84; 11.66) | 0.001 | 3.44 (1.22; 9.72) | 0.020 |
| **P for trend** |  | <0.001 |  | 0.247 |

* Adjusted for age, sex, waist-hip ratio, systolic blood pressure < vs. ≥120 mmHg, antihypertensive medication (renin-angiotensin-aldosterone system blockers, calcium channel blockers, betablockers), lipid-lowering medication, HbA1c, diabetes duration, eGFR strata at baseline, and hemoglobin level at baseline.

Supplemental Table S3: Subgroup analyses assessing the association between nocturnal hypoxemic burden and the incidence of very high risk CKD.

| **T90 Q4 vs. Q1/2/3** | OR (95% CI), univariable | P value | OR (95% CI), multivariable* | P value | P Interaction term |
| --- | --- | --- | --- | --- | --- |
| Age ≤ 65 y | 1.58 (0.55; 4.58) | 0.398 | 1.18 (0.37; 3.82) | 0.777 | 0.297 |
| Age > 65 y | **2.79 (1.57; 4.97)** | **<0.001** | **2.64 (1.38; 5.07)** | **0.003** |  |
| Female | **3.21 (1.28; 8.07)** | **0.013** | **2.84 (1.04; 7.75)** | **0.041** | 0.493 |
| Male | **2.35 (1.30; 4.22)** | **0.004** | 1.87 (0.94; 3.75) | 0.076 |  |
| T2D ≤ 5 y | **5.83 (1.75; 19.44)** | **0.004** | 3.38 (0.79; 14.51) | 0.102 | 0.344 |
| T2D > 5 y | **2.22 (1.28; 3.83)** | **0.004** | **1.88 (1.02; 3.48)** | **0.043** |  |

*Adjusted for sex, age, waist-hip-ratio, HbA1c, diabetes duration, hypertension (yes/no), eGFR strata at baseline. Not adjusted for variable stratified for.

Supplemental Table S4: Baseline characteristics of 1179 patients.

| **Variables** | **Entire cohort** |
| --- | --- |
| n (%) | 1179 |
| Female sex, n (%) | 482 (40.9) |
| Age [years] | 66 ± 9 |
| BMI [kg/m^2^] | 31.0 ± 5.4 |
| Waist-hip ratio | 0.95 ± 0.08 |
| Former or current smokers, n (%) | 664 (56.4) |
| High alcohol intake, n (%) | 334 (28.8) |
| Physical activity, n (%) | 507 (43.7) |
| Socioeconomic status, n (%) |  |
| Low | 333 (28.8) |
| Lower-middle | 591 (51.1) |
| Upper-middle | 147 (12.7) |
| High | 86 (7.4) |
| eGFR [**ml/min/1.73m²]** | 80 [64; 91] |
| eGFR category [ml/min/1.73 m²], n (%) |  |
| ≥90 (stage 1) | 338 (28.7) |
| 60-89 (stage 2) | 610 (51.7) |
| 30-59 (stage 3) | 212 (18.0) |
| <30 (stage 4/5) | 19 (1.6) |
| uACR [mg/g] | 10.3 [4.6; 30.3] |
| uACR category, n (%) |  |
| <30 mg/g | 859 (74.8) |
| 30-300 mg/g | 290 (25.2) |
| >300 mg/g | - |
| CKD risk stage, n (%) |  |
| Normal/low | 721 (62.8) |
| Moderate | 274 (23.8) |
| High | 102 (8.9) |
| Very high | 52 (4.5%) |
| HbA1c [mmol/mol (%)] | 49 (6.6) [44 (6.2); 55 (7.2)] |
| T2D duration [years] | 9.6 [5.4; 15.2] |
| Any glucose-lowering medication, n (%) | 1005 (85.2) |
| Insulin, n (%) | 346 (29.8) |
| Oral antidiabetic drug, n (%) | 890 (75.5) |
| Systolic BP [mmHg] | 139 ± 18 |
| Diastolic BP [mmHg] | 74 ± 10 |
| Cardiovascular conditions, n (%) |  |
| Hypertension | 494 (42.4) |
| Coronary artery disease | 221 (18.7) |
| Stroke | 66 (5.6) |
| Antihypertensive drugs, n (%) | 915 (77.6) |
| LDL [mg/dl] | 117 ± 35 |
| HDL [mg/dl] | 54 ± 16 |
| Lipid lowering drugs, n (%) | 577 (48.9) |
| Mean SpO_2_ [%] | 92 ± 2 |
| Min SpO_2_ [%] | 81 [78; 83] |
| T90 [%/TRT] | 3.6 [0.5; 13.6] |
| T90_non-specific_ [%/TRT] | 0.9 [0.0; 7.6] |
| T90_desaturation_ [%/TRT] | 1.5 [0.3; 5.0] |
| ODI [events/h] | 7 [3; 15] |
| AHI [events/h] | 10 [5; 19] |
| Excessive daytime sleepiness, n (%) | 82 (7.1) |
| PAP use at follow-up, n (%) | 122 (10.5) |
| Follow-up time [years] | 6.7 [6.1; 8.4] |

Results are provided as mean ± standard deviation or median [interquartile range].

AHI: apnea-hypopnea index, BMI: body-mass index, BP: blood pressure, CKD: chronic kidney disease, HbA1c: hemoglobin A1c, HDL: high-density lipoprotein, LDL: low-density lipoprotein, ODI: oxygen-desaturation index, PAP: positive airway pressure, SpO_2_: arterial oxygen saturation, T90: night-time spent with oxygen saturation <90%, uACR: **urinary albumin-to-creatinine ratio.**

High alcohol intake defined as ≥3/drinks per week; excessive daytime sleepiness defined as Epworth Sleepiness Scale ≥11; physical activity defined as light activity >2 times/week; hypertension defined as blood pressure ≥140/90 mmHg. Antidiabetic drugs including insulin, biguanides, sulfonylureas, glitazones, DPP-4 inhibitors, GLP-1 receptor agonists, SGLT2 inhibitors, alpha-glucosidase inhibitors.

Supplemental Table S5: Subgroup analyses assessing the association between nocturnal hypoxemic burden and the incidence of kidney failure.

| **T90 Q3/4 vs. 1/2** | **OR (95% CI), univariable** | **P value** | **OR (95% CI), multivariable*** | **P value** |
| --- | --- | --- | --- | --- |
| **Entire cohort** | 1.79 (1.25; 2.55) | 0.001 | 1.23 (0.85; 1.79) | 0.282 |
| **Age <= 65 y** | **2.79 (1.31; 5.94)** | **0.008** | **2.26 (1.01; 5.04)** | **0.046**** |
| **Age > 65 y** | 1.34 (0.87; 2.08) | 0.185 | 1.08 (0.67; 1.72) | 0.758 |
| **Female** | 1.72 (0.89; 3.34) | 0.106 | 1.48 (0.74; 2.96) | 0.264 |
| **Male** | 1.66 (1.05; 2.63) | 0.031 | 1.30 (0.78; 2.15) | 0.310 |
| **T2D <= 5 y** | 2.71 (0.98; 7.45) | 0.054 | 1.91 (0.61; 5.95) | 0.266 |
| **T2D > 5 y** | 1,58 (1.05; 2.37) | 0.027 | 1.25 (0.81; 1.94) | 0.314 |

*Multivariable analyses adjusted for sex, age, waist-hip-ratio, HbA1c, diabetes duration, hypertension (yes/no), eGFR at baseline. Not adjusted for variable stratified for.

**Interaction term p=0.111

Supplemental Table S6:

Binary logistic regression model assessing the association between T90 quartiles and the incidence of **very-high-risk** CKD during follow-up, each quartile compared to the lowest quartile 1 stratified by baseline CKD risk class:

| **Baseline risk class** | **Low risk (n=585)** | **P value** | **Intermediate / high risk (n=272)** | **P value** |
| --- | --- | --- | --- | --- |
| **Quartile 1** | 1 (Ref.) |  | 1 (Ref.) |  |
| **Quartile 2** | 4.52 (0.50; 40.90) | 0.179 | 0.92 (0.35; 2.44) | 0.871 |
| **Quartile 3** | 5.89 (0.68; 51.04) | 0.107 | 0.75 (0.28; 2.02) | 0.753 |
| **Quartile 4** | 5.50 (0.61; 49.83) | 0.130 | 2.01 (0.86; 4.70) | 0.108 |
| **P for trend** |  | 0.116 |  | 0.056 |

Supplemental Figures

Supplemental Figure S1: Study flow chart


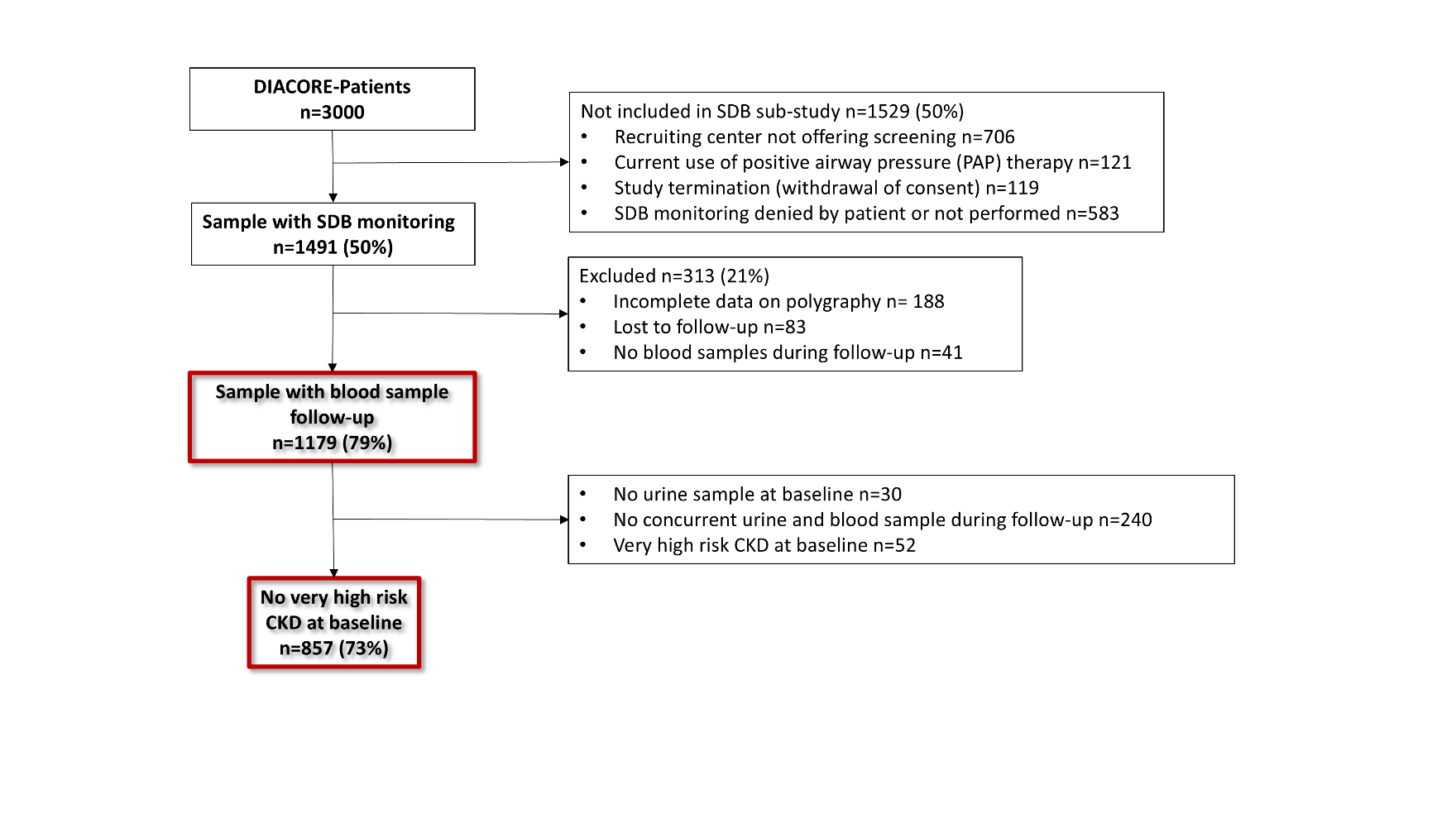

Supplement: Supplementary file 1 — Supplementary Material 1 [file 12933_2025_2918_MOESM1_ESM.docx]
